# Regulating glial morphogenesis: insights from *Drosophila*

**DOI:** 10.3389/fcell.2026.1775639

**Published:** 2026-03-26

**Authors:** Vrushali Katagade, Anuradha Ratnaparkhi

**Affiliations:** MACS-Agharkar Research Institute (Affiliated to Savitribai Phule Pune University), Developmental Biology Group, Pune, Maharashtra, India

**Keywords:** cell shape regulation, *Drosophila*, glia, GPCR, morphogenesis, neuro-glia

## Abstract

Glia are critical cellular components of the nervous system. Their morphogenesis during development is an important process that ensures formation of a functional nervous system in the adult organism. *Drosophila melanogaster* has been used extensively as a model to study the cellular and molecular mechanisms underlying glial development primarily because of the presence of different types of glia that are functionally analogous those found in vertebrates. In this review, we summarize and discuss signalling pathways that drive glial morphogenesis in *Drosophila* with a focus on those that regulate shape during development. We systematically discuss the different types of glia, their origin, function and signalling mechanisms that operate to regulate the ‘form-function’ relationship across development. We conclude by drawing attention to questions that need to be addressed, and the signalling pathways that need to be explored in this context which has implications to both, development and disease.

## 1 Introduction

Glia play a crucial role in sculpting and regulating function in the nervous system. Some of their key functions include serving as guidepost cells during circuit formation, compartmentalizing the nervous system into functional units and regulating neuronal metabolism (De Backer and Kadow, 2022; Coutinho-Budd et al., 2024). Another major function is neuroprotection: glia function as immune cells to stave off infections and promote healing upon injury (Allen and Barres, 2009). These diverse functions are performed by different classes of glia albeit with some degree of functional redundancy. The vertebrate nervous system has four main classes of glia: astrocytes, oligodendrocytes, Schwann cells and microglia (Freeman and Doherty, 2006). Each class has a distinct morphology with additional variations brought on by their position in the nervous system leading to significant diversity in shape. *Drosophila* glia are functionally analogous to those in vertebrates and therefore serve as an excellent model system to understand many aspects of glial biology. Each glial subtype is associated with a defined position and morphology determined during development to support their specific roles in the mature central nervous system (CNS) (Freeman and Doherty, 2006; Hartenstein, V. 2011; Freeman M.R. 2015; Corty and Coutinho-Budd, 2023; Awasaki and Lee, 2011). Loss of form affects function and thus understanding mechanisms that regulate glial shape is crucial for understanding structure-function relationships in glia and their impact on nervous system homeostasis.

In this review, we discuss signaling mechanisms regulating morphogenesis of each glial subtype. While differentiation establishes glial identity, morphogenesis encompasses processes that occur post differentiation such as growth, migration and cell shape change which affects tiling and organization. We begin each section with a short primer on mechanisms regulating differentiation followed by a comprehensive review of morphogenesis. We conclude with a section on ‘open questions’ where we discuss some key areas and questions that need to be addressed.

## 2 Glial diversity in *Drosophila*

In *Drosophila*, glia are generated in two waves: the first is during embryonic development, giving rise to the larval nervous system; the second wave occurs in the larva and generates most of the neurons and glia required in the adult, although many cells from the embryonic CNS are also retained in the adult (Omoto et al., 2015).

**FIGURE 1:**
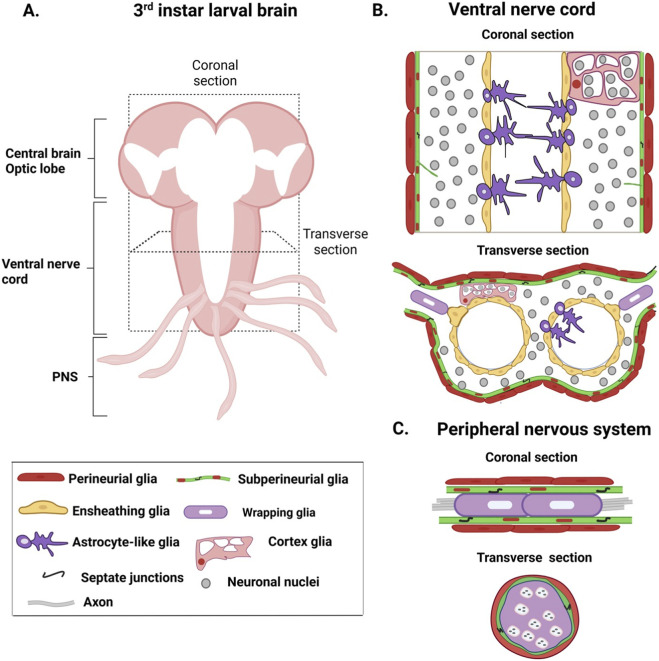
Organization of glia in the central and peripheral nervous system. **(A)** A schematic of a third instar larval brain indicating the coronal and transverse sections. **(B)** Coronal and transverse section of the ventral nerve cord showing organization of different glia: the subperineurial and perineurial glia constitute the outermost layer; astrocyte-like glia (ALG) extends processes into the neuropil; ensheathing glia enwrap the neuropil region. Cortex glia ensheath neurons present in the cortical layers. **(C)** Coronal and transverse section of the peripheral nerve showing organization of surface and wrapping glia.

Glia are broadly grouped into three classes namely neuropil, cortex, and surface glia. The neuropil glia (NPG) reside at the interface of the cortex and neuropil. This class includes the astrocyte-like glia (ALG) and ensheathing glia (EG). ALGs, like mammalian astrocytes, extend processes into the neuropil and branch extensively resulting in fine processes that surround synapses. They regulate neuronal and synaptic signaling and also perform phagocytic functions during development. EGs on the other hand, extend flat sheath-like processes around the neuropil and some major axon tracts to help compartmentalize the cortex from the neuropil, and different regions of the neuropil from each other. Cortex or cell body glia (CG) are located in the cortical regions populated by the neuronal soma. These glia have a small cell body that extends multiple processes to surround individual neurons. Surface glia constitute the outermost glial layer of the central and peripheral nervous system. This class includes (i) the outer perineurial (PG) which are flat cells with complex filopodia-like extensions, and (ii) the subperineurial glia (SPG) which are large, polygonal cells that tile with each other through septate junction to form a sheath that constitutes the blood-brain barrier (BBB) (Doherty et al., 2009; Hartenstein, V. 2011). The peripheral nervous system is ensheathed and protected by two classes of glia: the inner peripheral ensheathing glia and the outer surface glia consisting of subperineurial and perineurial glia that enwrap nerve bundles to constitute the blood-nerve barrier (BNB) (Banerjee and Bhat, 2007; Stork et al., 2008; Figure 1).

## 3 Development of neuropil glia in the CNS

**FIGURE 2:**
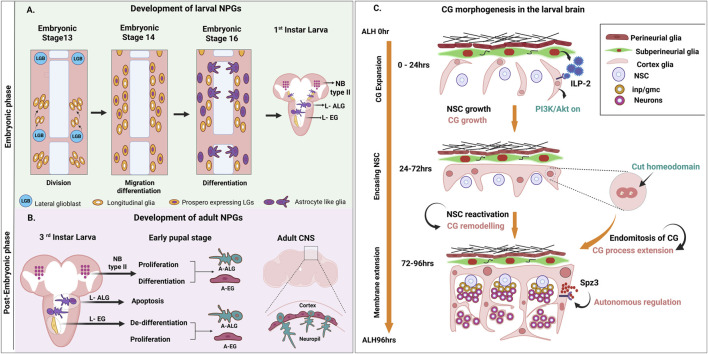
Development of neuropil and cortex glia. **(A)** Development of larval NPGs. These cells arise from division of a pair of lateral glioblasts (LGB) present in each segment of the embryonic CNS. Prospero positive LG (Purple nucleus) give rise to larval ALGs (L-ALGs) while the others (white nucleus) develop into larval EGs (L-EGs). **(B)** Development of Neuropil glia in the adult CNS. ALGs and EGs in the adult brain arise through two independent mechanisms: (i) From division and differentiation of type II neuroblasts and (ii) through de-differentiation of EG followed by re-differentiation into ALGs and EGs. **(C)** Morphogenesis of CGs in the larval brain. These glia cells, positioned near neural stem cells (NSCs), respond to non-autonomous signals like ILP-2 and autonomous factors namely Cut and Spätzle leading to extension of glial processes necessary for encasement of individual neuroblast lineages.

Neuropil glia (NPG) in the embryonic CNS are interchangeably referred to as longitudinal glia (LG) or interface glia (IG). They are positioned dorsal to the neuropil and constitute a cluster of 9–10 cells per hemisegment. Initial studies identified LGs as a subset of IGs arising from a lateral glioblast (Jacobs et al., 1989; Doe et al., 1991). However, DiI labelling studies have now firmly established that all the 9–10 IGs present per hemisegment are indeed LGs produced from the single lateral glioblast. Further, this lineage is heterogeneous, with subsets of glia expressing transcription factors Prospero and Fushi-Tarazu (Ftz) (Jacobs et al., 1989; Beckervordersandforth et al., 2008). Towards the end of embryogenesis, LGs occupy fixed positions around the neuropil and get specified into primary ALG and EG (Omoto et al., 2015; Figure 2A,B). In the adult, NPGs arise anew through division of typeII neuroblasts (Omoto et al., 2015; Viktorin et al., 2011), and also from some larval EGs that persist through metamorphosis by dedifferentiating and redifferentiating to give rise to both, EGs and ALGs (Kato et al., 2020; Figure 2A,B). Interestingly, in response to neuronal injury, EGs can also switch identities, differentiating into ALGs and transdifferentiating into neurons to promote healing (Casas-Tinto et al., 2025).

### 3.1 Mechanisms regulating morphogenesis of neuropil glia

The embryonic precursors of larval NPGs are the longitudinal glia (LG) which are arranged in two columns on either side of the midline. Organization of LGs is regulated by Fibroblast Growth Factor Receptor/Heartless (FGFR/Htl) signaling: In FGFR/Htl mutants, the LGs appear less closely packed and more round in shape (Campbell et al., 1994; Shishido et al., 1997). A similar phenotype is seen upon knockdown of *folded gastrulation* (*fog*) which encodes a secreted ligand that activates Gα_12/13_ (Concertina) through G-protein Coupled Receptors (GPCRs) Smog and Mist to trigger apical constriction during gastrulation (Costa et al., 1994; Morize et al., 1998; Dawes-Hoang et al., 2005; Manning and Rogers, 2014; Kerridge et al., 2016). Overexpression of Fog in LG causes ectopic glial midline crossing leading to disruption of the glial lattice. Interestingly, this phenotype is completely suppressed by loss of *concertina* indicating not only conservation of the signaling pathway, but also the importance of G-protein signaling in LG organization (Ratnaparkhi and Zinn, 2007; Shweta K et al., 2021). The involvement of G-protein signaling is supported by early studies that identified Loco (Regulator of G-protein Signaling (RGS)) as a regulator of LG organization and axonal ensheathment (Granderath et al., 1999).

A network of transcription factors regulate Loco expression in the LG. Activation in early embryonic CNS is controlled by Glial cells missing (Gcm), the master regulator of glial development, while Pointed (Pnt) and Deadringer (Dri) activate loco at a late stage (Granderath et al., 2000; Shandala et al., 2003). Not surprisingly, *pnt* and *dri* mutants show disorganized LG, pointing towards a regulatory network underlying glial organization.

Towards the end of embryogenesis, LGs differentiate into ALGs and EGs. FGFR/Htl signalling is a key regulator of ALG and EG morphology. In ALGs, FGFR/Htl signaling regulates arbor complexity by influencing density and length of the astrocytic processes. The pathway is activated by neuron derived Pyramus and Thisbe with latter being more a potent stimulator of arbor growth (Stork et al., 2014). A decrease in arbor growth has functional consequences, affecting phagocytosis of apoptotic neurons during late embryogenesis (Ayoub et al., 2023). A similar neuro-glial cross-talk involving FGFR/Htl signaling is also seen in case of EGs of the adult antennal lobe where Thisbe secreted by olfactory receptor neurons serves as an instructive signal to regulate ensheathment by EGs. Here, loss of signalling leads to poor compartmentalization of antennal glomeruli (Wu et al., 2017). The neuron-glia communication seen in the context EGs and ALGs bears resemblance to the vertebrate system where neuron-secreted Sonic Hedgehog (Shh) regulates astrocyte morphogenesis. In this context, expression of Shh-dependent genes such as Lrig1 in cortical astrocytes positively regulates arbor complexity (Xie et al., 2022).

In contrast to FGFR/Htl, the role of G-protein signaling in regulating EG and ALG morphology has remained largely unexplored. A recent study by Chen and colleagues identified GPCR Tre-1 as an autonomous regulator of ALG morphology whose functional analog in zebrafish is also found to regulate astrocyte complexity (Chen et al., 2024). Interestingly, expression of Fog, which activates G-protein signalling, is enriched in ALG precursors (Katagade et al., 2024) suggesting a likely role for the protein in regulating arbor formation. Further, FGFR/Htl functions to restrict Fog signaling in LGs: the ectopic glial midline crossing in response to Fog overexpression is enhanced by loss of FGFR/Htl. This suggests that a crosstalk between the two pathways is important in regulating LG organization and possibly morphology as well (Shweta K et al., 2021).

ALGs associated with the lamina, medulla and lobular regions of the visual system exhibit more varied and complex morphologies (Edwards et al., 2012) which parallels the mouse cortex where astrocytes show layer-specific morphologies regulated by neuro-glial interactions (Lanjakornsiripan et al., 2018). However, little is known of the molecular mechanisms underlying the formation of these complex shapes. Richier and colleagues have identified Lapsyn, a Leucine Rich Repeat containing transmembrane protein as a factor regulating positioning and morphology of ALGs of the medulla neuropil glia (Richier et al., 2017). However, the function of Lapsyn is not restricted to these cells, but extends to other ALGs as well (Richier B et al., 2017).

Both, ALGs and EGs exhibit plasticity in shape that enables them to adapt and respond to changes in their environment. During metamorphosis, EGs and ALGs undergo dynamic cell shape change essential for phagocytosis of apoptotic neurons and other neuronal debris (Hakim et al., 2014; Tasdemir-Yilmaz and Freeman, 2014; Hilu-Dadia and Kurant, 2020). This is also seen upon injury when EGs extend processes to invade the neuropil space and phagocytose axonal debris-a process dependent on Draper, the engulfment receptor (MacDonald et al., 2006; Doherty et al., 2009; Ziegenfuss et al., 2012). The cell shape change underlying phagocytosis is mediated by a guanine nucleotide exchange factor (GEF) complex Crk/Mbc/dCed-12 and Rac1 which controls actin remodelling (Ziegenfuss et al., 2012). The same complex functions in astrocytes to regulate phagocytosis of mushroom body neurons during metamorphosis (Tasdemir-Yilmaz and Freeman, 2014).

Collectively, these studies identify FGFR/Htl and G-protein signaling as important regulators of EG and ALG positioning during embryonic development. They also establish neuro-glial interactions as key regulators of glial shape.

## 4 Development of cortex glia

Cortex glia (CG) occupy the cortical regions of the CNS. In the embryo, these glia arise from division of neuroblasts (Ito et al., 1995) after which they migrate to their defined positions in the ventral nerve cord (Beckervordersandforth et al., 2008). By stage 16, they assume a radial morphology, extending processes throughout the cortical region of the CNS to envelope individual neuronal cell bodies creating a mesh-like network towards the end of embryogenesis. The density of this network increases after hatching so that by second instar every neuronal cell body is enveloped by CGs with each CG enwrapping multiple neurons (Pereanu et al., 2005). CGs provide metabolic support and protect neurons by preventing abnormal synchronous neuronal excitability through regulation of intracellular Ca^2+^ oscillations (Melom and Littleton, 2013; Volkenhoff et al., 2015).

### 4.1 Mechanisms regulating morphogenesis of cortex glia

Post embryogenesis, morphology of cortex glia undergoes a dramatic change in the period between 48 and 96 hours after larval hatching (ALH), the number of cortex glia increases exponentially. FGFR/Htl signaling activated by neuronally derived Pyramus plays a crucial role in the proliferation of CGs (Avet-Rochex et al., 2012). Interestingly, while some CGs divide completely through mitosis, others undergo endoreplication or acytokinesis resulting in polyploid or syncytial, multinucleate glia respectively (Rujano et al., 2022). Endoreplication is found to be crucial for glial growth and is regulated by Cut, a transcription factor whose loss results in fewer glial extensions affecting neuronal wrapping (Yadav et al., 2024; Figure 2C). Interestingly, an intronic enhancer in *fog* regulated by Abdominal-A, drives expression in CGs (Katagade et al., 2024) suggesting a possible role for Hox and segment identity genes in regulating CG morphogenesis.

Curiously, the initiation of CG growth in the larva coincides with the reactivation of neuroblasts, enabling individual neuroblasts and their progeny to be efficiently encased into separate lineages (Pereanu et al., 2005; Yuan et al., 2020; Rujano et al., 2022). The expansion of membrane essential for this extensive encasing is nutrient dependent and involves activation of PI3Kinase (Yuan et al., 2020; Figure 2C). In the brain lobe, this is dependent on Insulin-like peptide-2 (ILP-2) secreted by insulin producing cells while in the ventral nerve cord, the process is regulated by Insulin-like peptide 6 (ILP-6) secreted by the BBB which, interestingly, is independent of the canonical TOR-Akt pathway (Speder and Brand, 2018; Yuan et al., 2020).

The other factors associated with membrane expansion includes regulators of membrane composition and fusion. In ceramide phosphoethanolamine synthase mutants, altered plasma membrane composition results in compromised encapsulation of neurons leading to photosensitive epilepsy (Kunduri et al., 2018). Further, knockdown of genes regulating membrane fusion, *αSNAP*, *NSF2* and *Syntaxin5*, is found to result in atypical CG morphology (Coutinho-Budd et al., 2017). Spätzle (Spz3), a secreted neurotrophin, is another factor identified as an autonomous regulator of membrane growth. Loss of this gene impairs neuronal ensheathment leading to increased neuronal cell death (Coutinho-Budd et al., 2017). In conclusion, based on studies thus far, proliferation of CGs during the larval stages is non-autonomously regulated by FGFR/Htl signaling while morphology is influenced by nutrition and by autonomous factors affecting endocycling, membrane growth and fusion.

## 5 Development of surface glia

Surface glia includes perineurial (PG) and subperineurial glia (SPG). In the adult brain, these cells constitute 14% and 4% of the total glial population respectively (Kremer et al., 2017). The PGs lie below the neural lamella and form the outer layer while SPGs constitute the inner layer that ensheaths the entire brain to form the blood-brain barrier (BBB) (Awasaki et al., 2008; Stork et al., 2008). In the embryo, SPGs arise from neuroblasts present in the VNC (Ito et al., 1995; Schmidt et al., 1997). They subsequently migrate to the surface and grow synchronously to contact each other and establish septate junctions, forming a sealed sheath towards the end of embryogenesis (Schwabe et al., 2005). Thus, unlike vertebrates where the BBB is a complex, highly vascularized structure formed by endothelial cells, pericytes and astrocyte end-feet (Daneman and Prat, 2015; Sweeney et al., 2018), in *Drosophila*, the barrier is purely glia-based. Once ensheathment is complete, the SPGs continue to grow in size to accommodate the expanding volume of the larval brain (Schwabe et al., 2005).

Curiously, SPGs in the larval brain lobes are largely multinucleate polyploid cells while those in the ventral nerve cord and periphery are polyploid mononucleate cells indicating heterogeneity within the SPG population which causes some to continue with endoreplication while others undergo endomitosis (Unhavaithaya and Orr-Weaver, 2012). Notch signaling has been identified as a regulator of the switch between endoreplication and endomitosis although the molecular mechanisms governing these decisions is still poorly understood. Polyploidy however, appears to be essential for barrier integrity as inhibition of endoreplication or endomitosis leads to smaller SPGs and a compromised BBB (Von Stetina et al., 2018). Interestingly, SPGs are also found to extend fine processes to establish connectivity with PGs and NPGs (Schwabe et al., 2005; Contreras et al., 2024). The functional importance of this interconnectivity is not well studied.

### 5.1 Mechanisms regulating morphogenesis of subperineurial glia

Co-ordinated growth and cell morphology is crucial for the formation of the BBB. Moody, a GPCR, was amongst the first signaling molecules to be identified as a regulator of SPG morphology and BBB formation (Bainton et al., 2005; Schwabe et al., 2005). Consequently, most studies have centered around the role of Moody signalling in this process. A detailed study by Schwabe and colleagues shows that loss of *moody* results in uncoordinated growth leading to variability in size and shape of the SPGs. G-proteins, namely Gα_i/o,_ signal downstream of Moody and, together with RGS Loco, positively regulate barrier integrity albeit to varying degrees. Interestingly, sequestration of the βγ subunits has a more severe effect on BBB integrity indicating that this arm of GPCR signaling has a crucial role in barrier formation (Schwabe et al., 2005). The details of this regulation however, are still poorly understood.

Moody also regulates formation of actin rich structures (ARS) and localization of myosin along the SPG boundary (Hatan et al., 2011). This process is essential for maturation of septate junctions and maintenance of barrier integrity (Hatan et al., 2011; Babatz et al., 2018). Curiously, despite its role in BBB formation, *moody* mutant embryos are able to hatch (Schwabe et al., 2005) and the insulation defects are less severe compared to *loco* mutants indicating a likelihood of other GPCRs being involved in BBB formation. In this context, it is interesting to note that although Tre-1 is expressed in SPGs, it has no effect on the BBB (Schwabe et al., 2005).

Interestingly, the ligand Fog is also expressed in SPGs (Katagade et al., 2024). Given its role in regulating actomyosin in epithelial cells (Parks and Wieschaus, 1991; Morize et al., 1998; Dawes-Hoang et al., 2005), it is likely that Fog in SPGs functions similarly to regulate actomyosin organization essential for BBB formation. Interestingly, Fog has recently been shown to signal via Gα_q_ in neural stem cells (Lin et al., 2024). This versatility in terms of the downstream G-protein opens up the possibility that Fog may signal via multiple GPCRs, including Moody, to potentially influence SPG morphogenesis and BBB formation.

## 6 Open questions

Studies in vertebrate systems have shown that FGFR signaling plays a crucial role in regulating glial morphogenesis (Furusho et al., 2011; Furusho et al., 2012; Meier et al., 2014; Kang et al., 2014). This appears to be the case in *Drosophila* as well: FGFR/Htl signaling is found to regulate organization, morphology and plasticity in most classes of glia across developmental stages (Shishido et al., 1997; Avet-Rochex et al., 2012; Stork et al., 2014; Ayoub et al., 2023). On the other hand, little is known of how G-protein signaling pathways regulate glial morphology. Much of our understanding comes from studies on Moody which is limited to SPGs. However, studies on Loco, Fog and Tre-1 (Granderath et al., 1999; Schwabe et al., 2005; Ratnaparkhi and Zinn, 2007; Shweta et al., 2021; Chen et al., 2024; Katagade et al., 2024) indicate that GPCR signaling is likely to have a broader role in regulating glial morphogenesis which needs to be explored further. In this context, the interaction between Fog and FGFR/Htl in regulating glial organization (Shweta et al., 2021) is significant especially given the conserved role of FGFR in glial morphogenesis. It would be interesting to determine if the interaction between the two pathways (GPCR and FGFR/Htl) is conserved and hierarchical in other glia as well.

Studies show that neuro-glial interactions play a crucial role in shaping glial morphology. This is not surprising since the two cell types lie in close proximity to each other. Indeed, activation of FGFR/Htl signaling in NPGs and CGs is non-autonomous with neurons being the source of the ligand. The importance of non-autonomous signals is supported by a recent study of Lago-Baldaia and colleagues. The authors, through their single cell transcriptomic study, show that barring a few exceptions, morphological variations are not supported by variations in the transcriptome, indicating that non-autonomous inputs have a major role to play in determining glial architecture (Lago-Baldaia et al., 2023).

In view of this, it is surprising that very few factors have been identified that mediate neuro-glial interactions. One obvious set of molecules would be receptors and transporters associated with neurotransmission. This is seen in vertebrates where inhibiting mGluR in developing cortical astrocytes affects arborization (Morel et al., 2014). The lack of sufficient information on non-autonomous factors makes it imperative to focus on how neuro-glia and glia-glia interactions affect glial organization, shape and tiling.
